# Recent trends in revision knee arthroplasty in Germany

**DOI:** 10.1038/s41598-021-94988-7

**Published:** 2021-07-29

**Authors:** Markus Rupp, Nike Walter, Edmund Lau, Michael Worlicek, Steven M. Kurtz, Volker Alt

**Affiliations:** 1 Department of Trauma Surgery, University Medical Center Regensburg, Franz-Josef-Strauß-Allee 11, 93053 Regensburg, Germany; 2 Exponent Inc, Menlo Park, CA USA; 3 Exponent Inc, Philadelphia, PA USA

**Keywords:** Medical research, Epidemiology

## Abstract

We aimed to answer the following questions: (1) How did numbers of revision knee arthroplasty procedures develop in Germany over the last decade compared to primary TKA? (2) How high was the percentage of septic interventions in knee prosthesis revisions? (3) Which treatment strategy was chosen for surgical treatment of knee PJI? Revision arthroplasty rates as a function of age, gender, infection and type of prosthesis were quantified based on Operation and Procedure Classification System codes using **r**evision knee arthroplasty data from 2008 to 2018, provided by the Federal Statistical Office of Germany (Destatis). In 2018, a total number 23,812 revision knee arthroplasties were performed in Germany, yielding an overall increase of 20.76% between 2008 and 2018. In comparison, primary TKA procedures increased by 23.8% from 152,551 performed procedures in 2008 to 188,866 procedures in 2018. Hence, 12.6% of knee arthroplasties required a revision in 2018. Septic interventions increased by 51.7% for all revisions**.** A trend towards higher numbers in younger patients was observed. Compared to 2008, 17.41% less DAIR procedures were performed, whereby single-stage and two- or multi-stage change increased by 38.76% and 42.76% in 2018, respectively. The increasing number of revision knee arthroplasty in Germany, especially in younger patients and due to infection, underlines the need for future efforts to improve treatment strategies to delay primary arthroplasty and avoid periprosthetic joint infection.

## Introduction

The introduction of joint arthroplasty is one of the greatest achievements of modern medicine. Joint arthroplasty is one of the most efficacious and cost-effective surgical procedure. Since the 1960s, it has led to a significant reduction in pain and movement restrictions, thus significantly improving patients' quality of life^1,2^. This success and the demographic development in the industrialized nations have led to a significant increase in the number of total hip and total knee arthroplasties (TKA) which is predicted to continue in the next decades^3–6^. Especially an increasing number of primary knee arthroplasties in younger patients leads us to anticipate an increasing number of revision surgeries in the coming years. Recently, projections of revision knee arthroplasties based on numbers of past knee revision arthroplasties have been published^7–9^. For the U.S. an increase in revision TKA is predicted between 78 and 182% within the next 10 years^8^. Although these data suggest that the expected increase in revision arthroplasty is likely to occur due to the significant increase in primary TKAs, estimation of future data based on recent revision arthroplasty numbers is subject to significant limitations. For instance, the risk of periprosthetic joint infection (PJI) decreases with increasing prosthesis lifetime, while aseptic loosening increases^10,11^. In addition, the revision rate depends on the number of patients living with an endoprosthesis. Besides, patients´ mortality rate as well as prosthesis lifetime would have to be taken into account in order to make a reliable statement about future revision rates. Since these different aspects cannot be reconstructed with certainty while relying on a large number of registry data, a detailed analysis of recent numbers seems to be beneficial to estimate future demands and to foresee developments which could be influenced by adaption of prevention and therapeutic measures.

We have therefore aimed to answer the following questions for the Germany population: (1) How did numbers of revision knee arthroplasty procedures developed over the last decade compared to primary TKA? (2) How high was the percentage of septic interventions in knee prosthesis revisions? (3) Which therapy strategy (debridement antibiotics implant retention (DAIR), single-stage change or two- or multi-stage change) was chosen for surgical treatment of knee PJI?

## Material and methods

Revision knee arthroplasty data from 2008 to 2018 was provided by the Federal Statistical Office of Germany (Destatis) consisting of annual surgical procedures performed in medical institutions of all 16 German federal states. Surgery and procedure keys (Operation and Procedure Classification System codes) were used to identify all revision knee arthroplasties in patients aged 20 years or older, regardless of the underlying disease or injury. In particular, the Operation and Procedure Classification System code “5-823, revision, exchange and removal of an knee joint endoprosthesis “ was used (Table 1). A detailed breakdown of these data by age group and gender was performed. Surgical strategies were retrieved by evaluating the Operation and Procedure Classification System codes in combination with ICD-10 diagnosis codes. In particular, the ICD-10 code “T84.5, infection and inflammatory reaction by a joint endoprosthesis” was used for septic cases and “T84.04, mechanical complication of a joint endoprosthesis” for aseptic cases. To compare revision rates to numbers of primary TKA, the Operation and Procedure Classification System code “5–822, implantation of an endoprothesis of the knee joint” was used. Prevalence rates were calculated based on Germany’s historical populationaged 20 years or older provided by Destatis. Data were analyzed using the statistical software SPSS Version 26.0 (IBM, SPSS Inc. Armonk, NY, USA).

**Table 1:** Operation and procedure classification system code descriptions.

Operation and procedure classification system code	Description
5-823.0	Revision without exchange
5-823.1	Exchange of unicondylar prosthesis
5-823.10+5-23.11	Exchange of unicondylar prosthesis to unicondylar prothesis
5-823.19	Inlay exchange
5-823.1a + 5-823.1b + 5-823.1c	Exchange of unicondylar prosthesis to bicondylar surface replacement prosthesis
5-823.1d + 5-823.1e + 5-823.1f.	Exchange of unicondylar prothesis to femoral and tibial shaft-anchored prosthesis
5-823.1x	Exchange of unicondylar prosthesis to other
5-823.2	Exchange of bicondylar surface replacement prosthesis
5-823.5	Exchange patella replacement
5-823.7	Explantation of bicondylar surface replacement prosthesis
5-823.9	Explantation patella replacement
5-823.b	Exchange endoprosthesis with extended flexion capability
5-823.d	Explantation endoprosthesis with extended flexion capability
5-823.f	Exchange of bicompartmental partial joint replacement prosthesis
5-823.g	Explantation of bicompartmental partial joint replacement prosthesis
5-823.h	Exchange of endoprosthetic joint replacement without movement function
5-823.j	Explantation of endoprosthetic joint replacement without movement function
5-823.k	Exchange of femoral and tibial shaft-anchored prosthesis
5-823.m	Explantation of femoral and tibial shaft-anchored prosthesis

## Results

**Figure 1:**
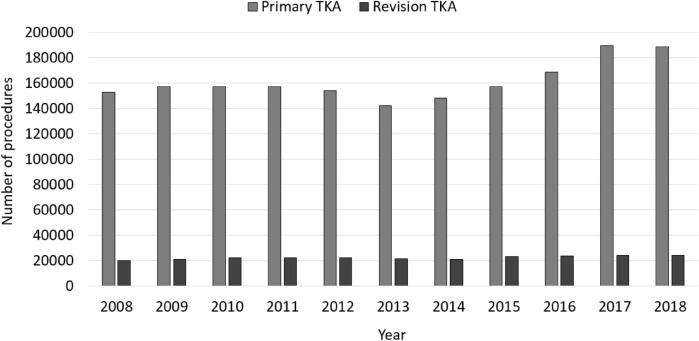
Performed revision knee arthroplasty procedures in comparison to performed primary knee arthroplasty procedures from 2008 through 2018.

In 2018, a total number 23,812 revision knee arthroplasties were performed in Germany, yielding an overall increase of 20.76% between 2008 and 2018 (Table 2). In comparison, primary TKA procedures increased by 23.8% from 152,551 performed procedures in 2008 to 188,866 procedures in 2018. Hence, 12.6% of knee arthroplasties required a revision in 2018 (− 0.3% change compared to 2008) (Fig. 1).

**Table 2:** Development of revision knee arthroplasty numbers.

Years	Performed revision knee arthroplasty	Prevalence per 100,000 inhabitants	Relative to 2008 (%)	Septic revisions	Male patients	Female patients	Patients younger than 65 years	Patients aged 65 years or older
2008	19,718	29.7		3402	7038	12,680	5554	14,164
				(17.25)	(35.69)	(64.31)	(28.17)	(71.83)
2009	20,789	31.3	5.43	3835	7705	13,084	5872	14,917
				(18.45)	(37.06)	(62.94)	(28.25)	(71.75)
2010	21,948	33	11.31	4035	8264	13,684	6445	15,503
				(18.38)	(37.65)	(62.35)	(29.36)	(70.64)
2011	22,316	34.1	13.18	4253	8410	13,906	6699	15,617
				(19.06)	(37.69)	(62.11)	(30.20)	(69.98)
2012	22,125	33.7	12.21	4110	8384	13,741	6739	15,386
				(18.58)	(37.89)	(62.11)	(30.46)	(69.54)
2013	21,454	32.5	8.80	4355	8353	13,101	6822	14,632
				(20.30)	(38.93)	(61.07)	(31.80)	(68.20)
2014	20,944	31.4	6.22	4291	8094	12,850	6657	14,287
				(20.49)	(38.65)	(61.35)	(31.78)	(68.22)
2015	23,151	34.5	17.41	5109	9104	14,047	7155	15,996
				(22.07)	(39.32)	(60.68)	(30.91)	(69.09)
2016	23,447	34.8	18.91	5060	9445	14,002	7395	16,052
				(21.58)	(40.28)	(59.72)	(31.54)	(68.46)
2017	23,772	35.4	20.56	5234	9516	14,256	7505	16,267
				(22.02)	(40.03)	(59.97)	(31.57)	(68.43)
2018	23,812	35.4	20.76	5161	9720	14,092	7518	16,294
				(21.67)	(40.82)	(59.18)	(31.57)	(68.43)

**Figure 2:**
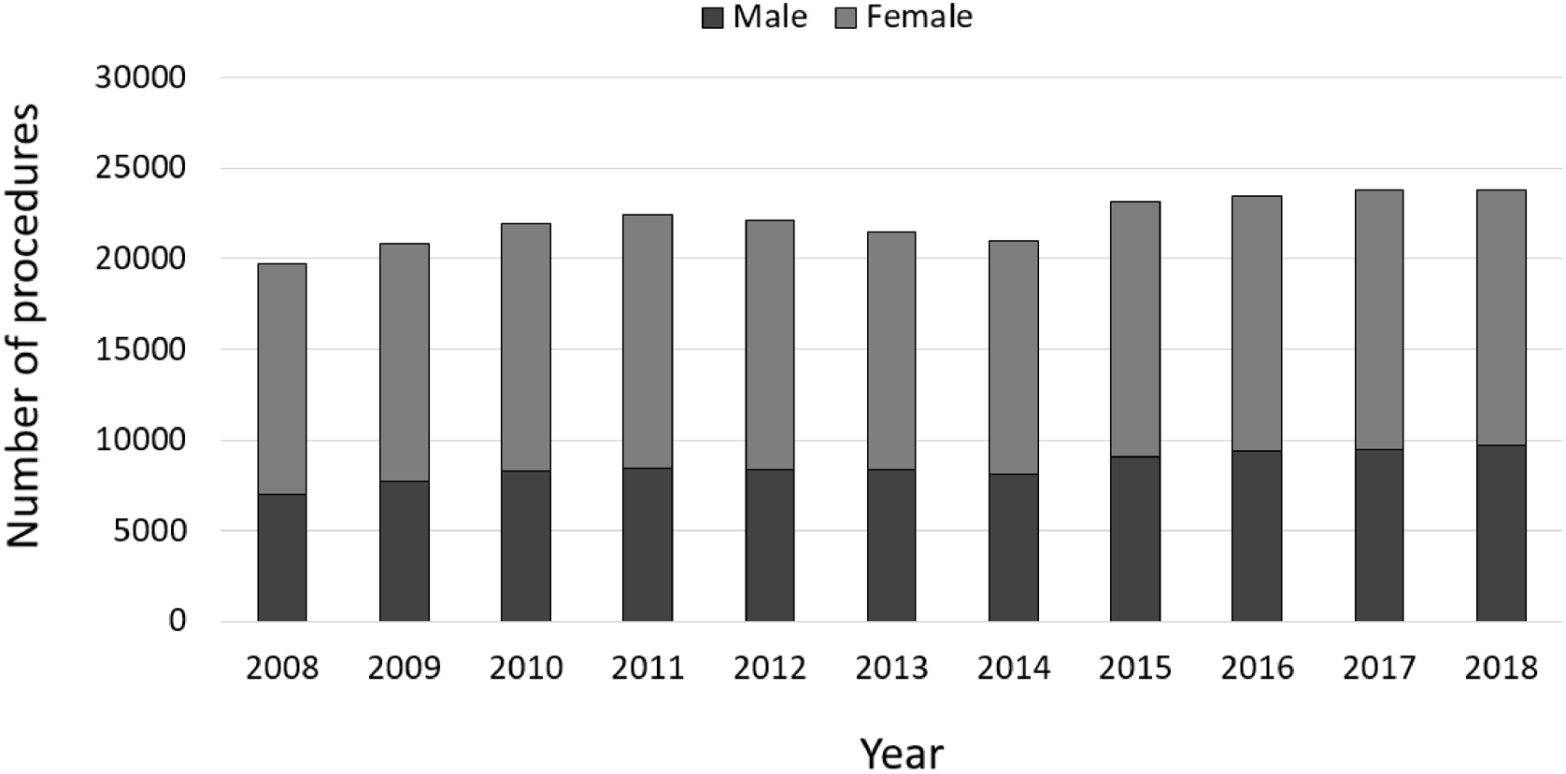
Performed revision knee arthroplasty procedures from 2008 through 2018 divided by gender.

**Figure 3:**
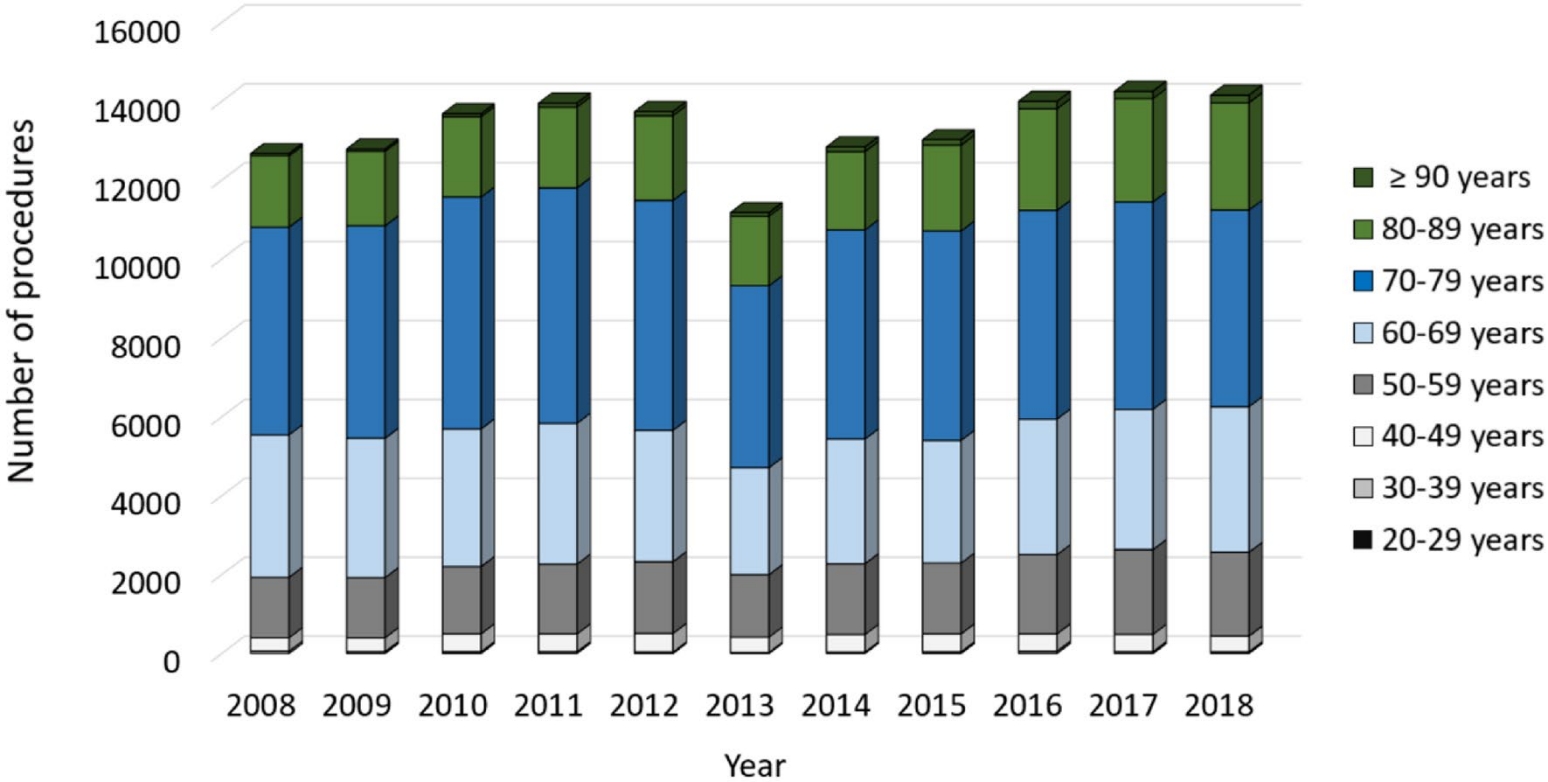
Revision knee arthroplasty procedures of female patients from 2008 through 2018 divided by age.

**Figure 4:**
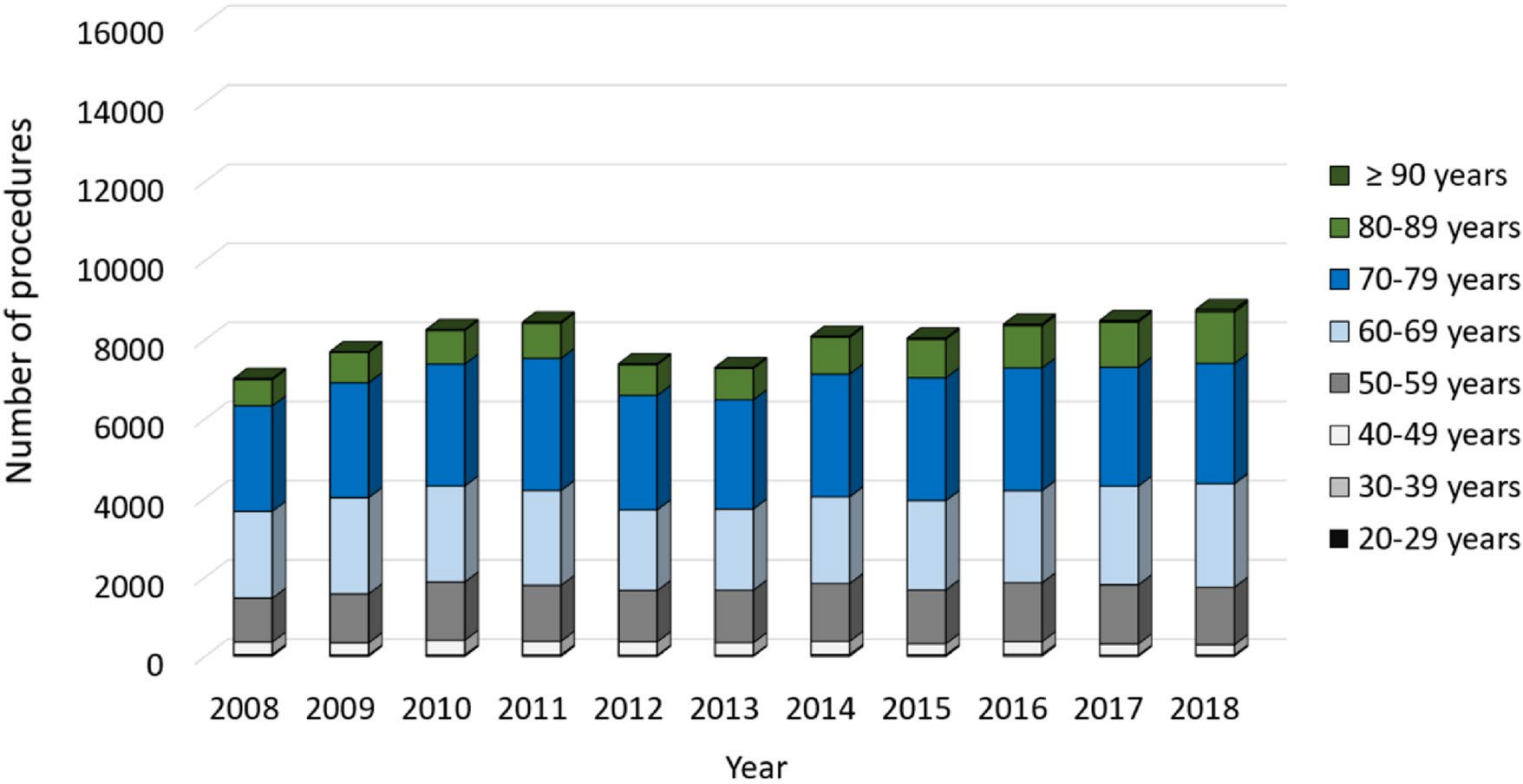
Revision knee arthroplasty procedures of male patients from 2008 through 2018 divided by age.

**Figure 5:**
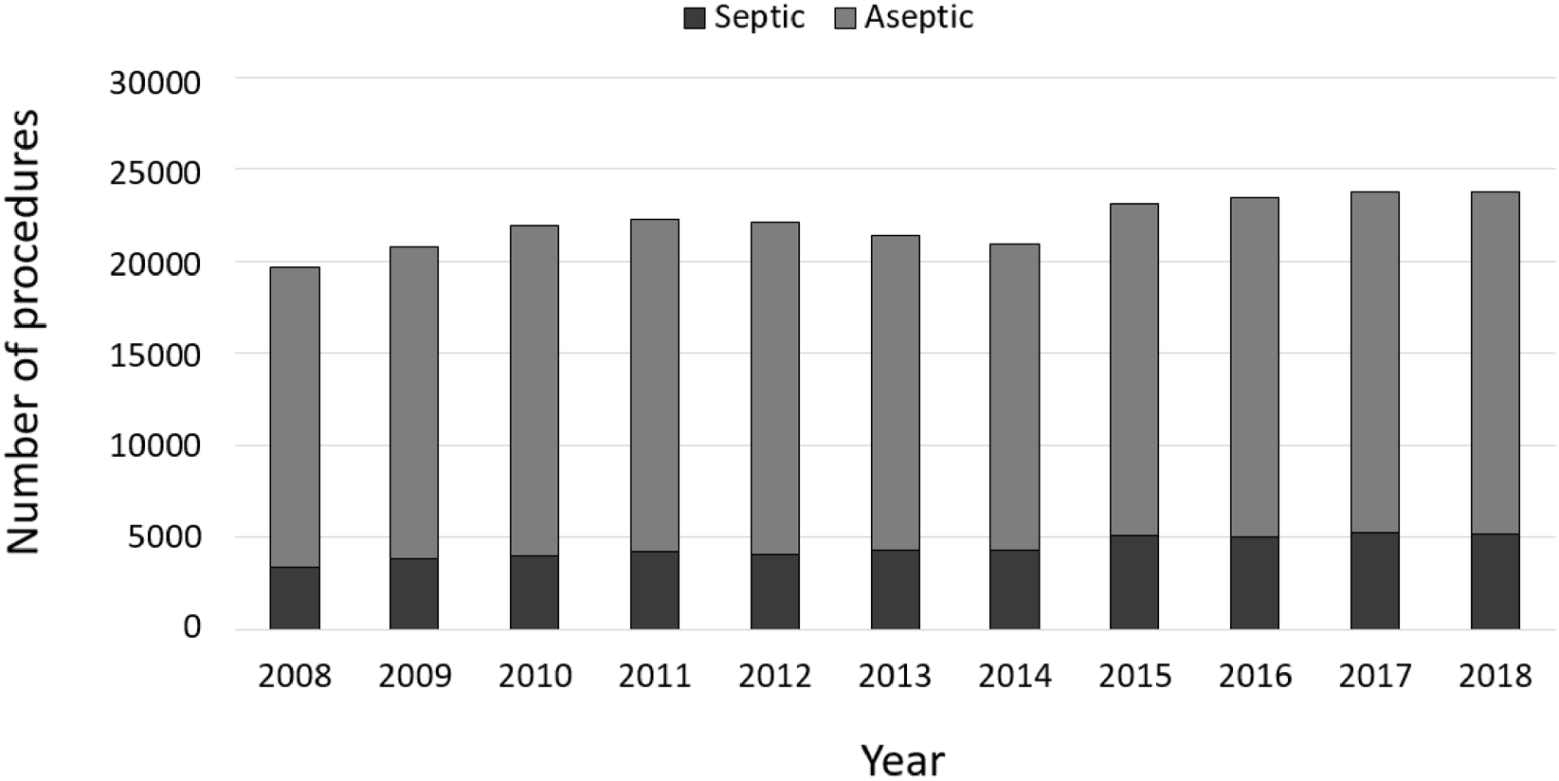
Performed revision knee arthroplasty procedures from 2008 through 2018 divided in septic and aseptic cases.

**Figure 6:**
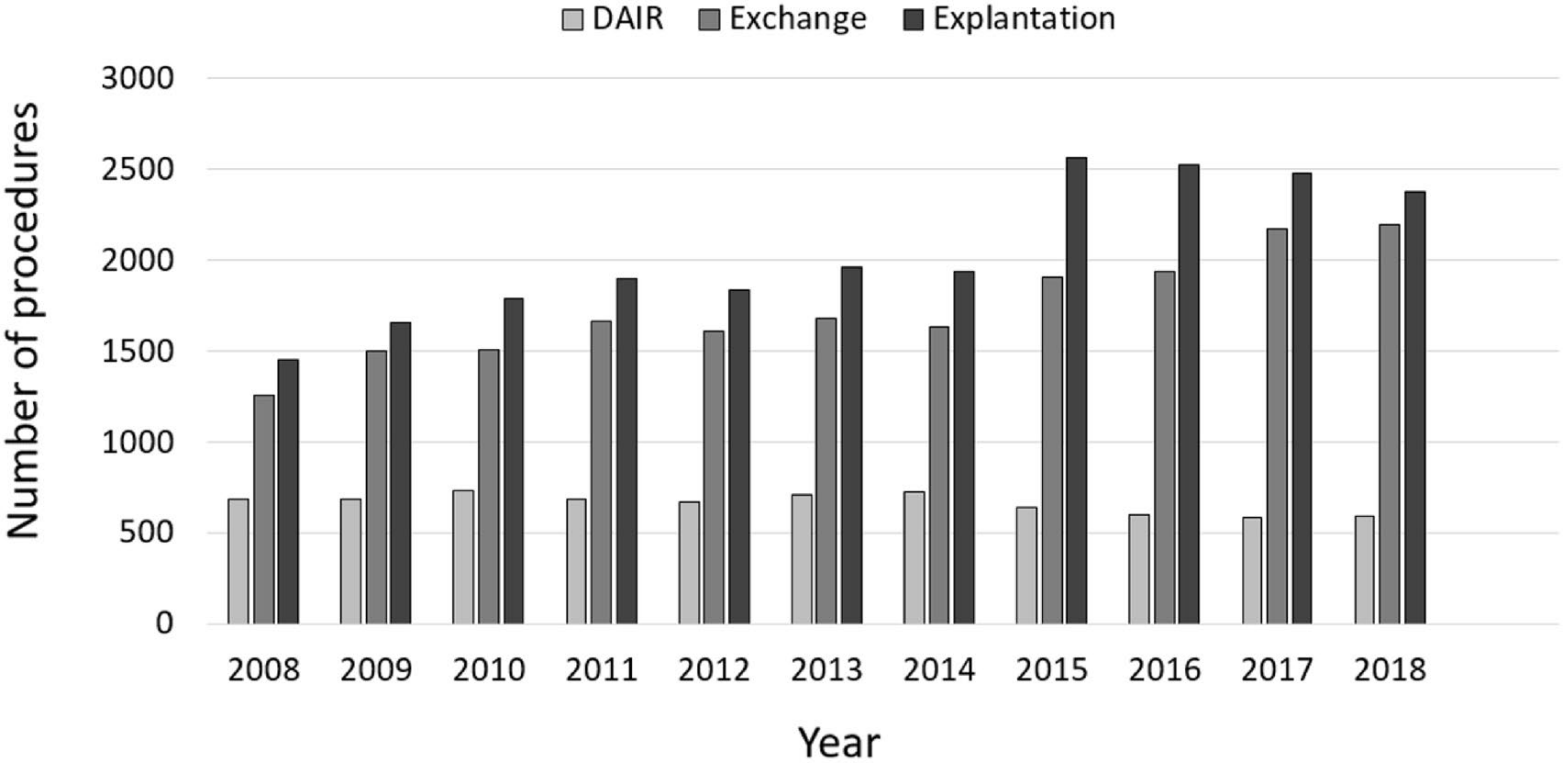
Performed surgical procedures in septic revision cases.

In general, more female than male patients were affected, whereas the numbers of male patients increased between 2008 to 2018 (Fig. 2). Patients aged 65 years or older comprised the largest cohort with 71.83% of all revision cases in 2008. Over time, a shift of the age distribution could be observed. Compared to ten years earlier, 1964 more revision knee joint endoprosthetic surgeries were performed on patients younger than 65 years in 2018, which depicts an increase of 7.37%. Most of revision procedures were carried out in patients aged 70–79 years (34.66% of all male patients and 35.23% of all female patients in 2018) followed by patients aged 60–69 years (29.84% of all male patients and 26.06% of all female patients in 2018) and patients aged 50–59 years (16.57% of all male patients and 15.01% of all female patients in 2018) (Figs. 3, 4). The rate of septic interventions in knee prosthesis revisions steadily increased by 51.7% over time from 3402 (17.25%) procedures in 2008 to 5161 (21.67%) procedures due to infection in 2018 (Fig. 5). In addition, the choice of treatment strategy changed over the years. In 2008, 20.28% of the PJI patients were treated with the debridement antibiotics implant retention (DAIR) approach (690 procedures), whereas 36.95% of the patients underwent a single-stage change (1257 procedures). 42.77% cases were managed with a two- or multi-stage change coded as “explantation” (1455 procedures). In 2018, however, DAIR procedures decreased to 11.41% (589 procedures), whereas the single-stage change as the chosen treatment strategy increased to 42.55% of all PJI cases (2196 procedures), and a two- or multi-stage change was performed in 46.04% of revisions due to infection (2376 procedures) (Fig. 6).

## Discussion

Our analysis outlines recent trends in revision knee arthroplasty from 2008 through 2018 in Germany. Total numbers of revision knee arthroplasty experienced a substantial overall increase during this period, similar to the increase of primary TKA numbers (+ 23.8% from 2008 through 2018). The revision burden (the quotient of revision and primary arthroplasty) in Germany (12.61% in 2018) compared to data reported for the US from 2005 through 2010 (9.1–9.6%, respectively) and comparisons between projected primary TKA numbers in the US and Germany make a detailed analysis of the revision knee arthroplasty numbers seem useful^3,4,12^.

### Influence of age, gender and primary arthroplasty procedure on revision TKA

A reported increase in revision TKA (rTKA) procedures of about 102% as published by Schwartz and coworkers between 2002 and 2014 could not be observed in Germany over a similar time frame^8^. The same applies to the analysis of different age groups. Schwartz et al. reported the largest increase in rTKA numbers in patients aging 55–64 years (+ 195% change) and those aged 65–74 years (+ 119% change). Our analysis could demonstrate highest increase in rTKA numbers in patients aged 90 years or older (+ 94.2% change), followed by patients aged 80–89 years (+ 63.0% change), which is by far less than the observed increase in revision numbers reported for the US. Despite a lower increase in rTKA, strategies to avoid revision arthroplasty should be intensified. Especially in geriatric patients who are extremely challenged by necessary surgical revisions with reported higher mortality rates after rTKA due to infection and fracture, efforts to avoid the causes for surgical interventions should be intensified^13,14^.

### Revision surgery for knee PJI accounts for about 20% of all cases

In accordance with formerly published analysis of revision TKA, revisions due to PJI account for about 20% of all cases. Bozic and coworkers analyzed reasons for rTKA in the US between 2005 and 2010 and reported PJI as reason for surgical revision to be 25%^12^. Other studies assessing reasons for reinterventions after primary TKA reported infection to be reason for rTKA in 30.3%^15^ and 20.3% of the cases, respectively^16^. An analysis from two orthopedic centers in Germany reported PJI accounting for rTKA from 14.5% through 26.8%, depending on time after index total knee arthroplasty^17^, which corresponds with reported higher incidence rates of rTKA due to PJI compared to aseptic loosening early after index TKA^10,18^.

### Septic revision TKA are increasingly managed by one-stage and two-stage exchange

Septic revision TKA are increasingly managed by one-stage and two-stage exchange (Fig. 6). Intriguingly, DAIR procedures did not show a substantial increase in numbers during the observation period. Analysis of operation and procedure codes for septic revision after TKA revealed a substantial increase of one-stage exchange as well as two-stage procedures. Reasons might be manifold. Since exchange arthroplasty should be performed in chronic PJI or acute PJI with implant loosening, DAIR procedures could be demonstrated to be reasonable in case of acute PJI either directly postoperative or in case of late hematogenous PJI^19^. Since our analysis allows not to distinguish between acute and chronic PJIs as rTKA reasons, a steady state in DAIR procedures would suggest chronic PJIs having increased over time or DAIR experienced less acceptance by the treating surgeons. A survey of 515 centers for primary and revision arthroplasty, however, revealed that DAIR is an established treatment alternative in 97.6% of the centers^20^. Thus, limited acceptance to DAIR might be due to factors associated with DAIR failure. Those are high numbers of patients´ comorbidities, infection with antibiotic resistant pathogens, duration of infection and previous revision surgeries^21^. In accordance with the increase of explantation procedures as a first step of a two-stage procedure, one-stage exchange experienced an increase in case numbers over the last 11 years. Defining recommendations for successful one-stage exchange arthroplasty might be a key driver for this trend^22^. Besides, favorable outcomes reported after one-stage exchange in chronic PJI might encourage orthopedic surgeons to save the patients a prosthesis-free interval with at least one more elaborate surgery^23^.

### Limitations

The study has several limitations. Historical inpatient data provided by Destatis have been analyzed based on OPS codes, which only allow distinction between different surgical procedures. Inherent limitation of all such analysis is the unverifiable accuracy of coding and data input. Since DRG lump sum reimbursement relies on accurate coding and reimbursement is strictly controlled by the Medical Service of Health Funds, correct coding of diagnosis and procedures can be assumed, however. Prosthesis types explanted and implanted could not be investigated by this approach. In addition, reasons for rTKA could not be itemized beyond revision due to infection using the unspecific ICD code T84.5 (revision arthroplasty due to infection). These downsides had to be accepted when analyzing 243,476 rTKA procedures. Since rTKA is generally performed as an inpatient procedure being reported to the German federal statistical office (Destatis), it can be assumed that the analyzed data set comprises all rTKA patients in the set time frame. Although, the investigated rTKA sample can be regarded as complete data set, patient characteristics additionally to gender and age, in general, have not been reported to Destatis, which unfortunately did not allow to analyze factors associated with type of rTKA. Additionally, no information about hospitals and their volume of rTKA was available which would be of interest in investigating application of treatment strategies and resource utilization.

## Conclusion

Although the increase in numbers of revision knee arthroplasty is smaller than observed for the US, increasing numbers of revision knee arthroplasty in younger patients and due to periprosthetic joint infection underlines the need for future efforts to improve treatment strategies to delay primary arthroplasty and avoid periprosthetic joint infection. Since other industrialized countries provide similar health care service and face similar demographic trends, the present analysis may help to adapt resource management for stakeholders in the health care systems worldwide.

## Data Availability

The datasets generated during and/or analysed during the current study are available from the corresponding author on reasonable request.
